# Exposure Assessment of Silver and Gold Nanoparticles Generated During the Synthesis Process in a South African Research Laboratory

**DOI:** 10.3389/ftox.2022.892703

**Published:** 2022-05-25

**Authors:** Masilu D. Masekameni, Charlene Andraos, Il Je Yu, Mary Gulumian

**Affiliations:** ^1^ Occupational Health Division, School of Public Health, University of the Witwatersrand, Johannesburg, South Africa; ^2^ N Toxicology and Biochemistry Department, National Institute for Occupational Health, National 7 Health Laboratory Services, Braamfontein, Johannesburg, South Africa; ^3^ HCT, Majang-myeon, Icheon, Korea; ^4^ Haematology and Molecular Medicine Department, University of the Witwatersrand, Johannesburg, South Africa; ^5^ Water Research Group, Unit for Environmental Sciences and Management, North-West University, Potchefstroom, South Africa

**Keywords:** OEL, SMPS, aitken, MPPD model, nanoparticles

## Abstract

During the synthesis of engineered nanomaterials (ENMs), various occupational exposures occur, leading to health consequences. To date, there is paucity of studies focused on modeling the deposition of nanoparticles emitted from ENMs synthesis processes. This study aimed to characterise and assess exposure to gold (AuNPs) and silver nanoparticles (AgNPs) during a synthesis process in a research laboratory in South Africa. AuNPs and AgNPs synthesis processes were monitored for an hour in a laboratory using a Scanning Mobility Particle Sizer. The monitoring was conducted at a height of 1.2–1.5 m (m) and 1.5 m away from the hood, assuming a 30 cm (cm) breathing circumference zone. Each synthesis process was monitored thrice to generate reliable point estimates, which were used to assess exposure over 8 hours. A time-weighted average concentration was calculated and compared to the derived 8-h occupational exposure limit (OEL) for AgNPs (0.19 μg/m^3^) and the proposed provisional nano reference value for AuNPs (20,000 particles/cm^3^). The Multiple-Path Particle Dosimetry model was used to calculate the deposition and retention of both AuNPs and AgNPs. NPs emitted during the synthesis process were dominant in the nuclei (79% for AuNPs and 54% for AgNPs), followed by the Aitken (12% for AuNPs and 29% for AgNPs), with fewer particles in the accumulation mode (9.2% for AuNPs and 17% for AgNPs). AuNPs and AgNPs generated during the synthesis process were determined at 1617.3 ± 102 cm^3^ (0.046 μg/m^3^) and 2,687 cm^3^ ± 620 (0.077 μg/m^3^), respectively. For the three exposure scenarios, none exceeded the occupational exposure limit for both AuNPs (provisional) and AgNPs (OEL). Workers in the synthesis laboratory are exposed to a concentration below the recommended occupational exposure limit for silver and the proposed provisional nano reference value for gold. Although, the concentrations to which laboratory workers are exposed to are below safe levels, the assessment of the lung deposition patterns indicate a high particle lung retention which raise concerns about long term safety of workers.

## 1 Introduction

Exposure to airborne aerosols in workplaces has been on the rise do to an increase in the utilisation of ENM’s for different applications in cosmetics, medical advancement and energy generation (Methner et al., 2010; Aldewachi et al., 2018). Several studies focusing on human health effects and respirable particles in workplaces have previously been conducted (Hobson and Guy 2014; Hoeflinger and Laminger 2019). Respirable particles are considered harmful to humans due to their chemical and physical properties (Murphy et al., 2008; Zhang and Tao 2009; Coradeghini et al., 2013). Since the 21st century, the use of engineered nanomaterials (ENMs) has gained immense interest in sectors such as medicine, industrial and domestic products (Botha et al., 2015; Corrêa et al., 2015; Ferdous and Nemmar 2020). ENMs can be better described as synthetic material possessing at least one size dimension between approximately one to 100 nm (Viet Long et al., 2013). There is compelling published literature on the human health risks associated with exposure to respirable particles with similar chemical properties to ENMs (Maynard and Kuempel 2005; Anwar and Halim 2012; Yahyaei et al., 2019; Hassanen et al., 2020).

Engineered nanoparticles (ENPs) are mainly released during synthesis, packaging, and use of products (Bauer et al., 2014; Hobson and Guy 2014; Pacheco et al., 2018). Several studies have been conducted in the manufacturing of nano-containing products, however, few studies focused on assessing exposure during the synthesis process (Sanabria et al., 2013; Chawla et al., 2018). Notably, gold nanoparticles (AuNPs) and silver nanoparticles (AgNPs) are used in various industrial and domestic applications (Akter et al., 2018). Silver ions are predominantly used in the water purification process, and to date, are still preferred in many more applications (Maurer and Meyer 2016). The advancement in the medical field has increased depending on the use of both AgNPs and AuNPs (Aldewachi et al., 2018).

ENPs can be released into the air during various production processes and downstream use stages and can rapidly enter the body through inhalation (Miller et al., 2017; Oberbek et al., 2019). Inhalation is the most common and harmful route of entry for many nanomaterials and those emitted as ENPs (Durantie et al., 2017). After exposure, some nanomaterials readily travel throughout the body, deposit in target organs, penetrate cell membranes, lodge in the mitochondria, and trigger injurious responses (Berardis and Marchetti 2020; Ferdous and Nemmar 2020). Some studies have demonstrated that ENPs can accumulate in the heart, liver, spleen, lungs, and kidneys (Cho et al., 2009; Abdelhalim and Moussa 2013; Kakakhel et al., 2021). Fewer studies have focused on the health effects associated with exposure to ENPs during the synthesis of ENMs in workplaces (Rodríguez-Ibarra et al., 2020). However, data generated from animal studies suggest that exposure to ENPs can lead to various health consequences ranging from cancer to non-cancer-related illnesses (Riaz Ahmed et al., 2017; Ferdous and Nemmar 2020).

Several dose metrics have been used to study the toxicity of ENPs and evidence suggests that the expression of dose in number or surface area of ENPs, rather than mass only, provides useful information to better understand exposure to disease evolution mechanisms (Methner et al., 2010). To date, various exposure assessment instruments and strategies have been proposed for monitoring nano-aerosol exposure in the workplace to assess mass, particle number or surface area (Kuhlbusch et al., 2018). Although there are health consequences associated with exposure to ENPs in the workplace, various ENMs have not yet been assigned occupational exposure limits (OELs). Instead, the United States National Institute for Occupational Safety and Health (NIOSH) has published OELs for certain ENPs. For example, a recommended exposure limit (REL) of 0.9 μg/m^3^ as an airborne respirable 8-h TWA concentration for AgNMs have been set (NIOSH 2021). In the absence of an established OEL for AuNPs, a proposed provisional nano reference value (NRV), which is based on a precautionary principle, has been set at 20,000 particles/cm^3^ by the National Institute for Public Health and the Environment and the Working Conditions Committee of the Social and Economic Council of the Netherlands (Jalava et al., 2006; Schwarze et al., 2006).

OELs are key indicators in exposure assessment, where a derived value can be used to rate the relative safety risk of external exposure dose. Information on nanoparticle deposition, retention and clearance maybe an important risk evaluation indicators of human exposure to silver and gold synthesis emissions. However, the toxicity of ENPs, as shown in several *in vivo* toxicological studies, focuses largely on the dose at the target organ of exposed animals. What remains a concern is the extrapolation of animal data to humans. Rats are primarily used in toxicity based studies and later the data is extrapolated to humans (Kim et al., 2020). Notably, rats and human deposited dosage were studied using mathematical models (Ji and Yu 2012). The use of the Multiple-Path Particle Dosimetry (MPPD) model has shown similarities in the deposition coefficient of ENPs (Buckley et al., 2016). However, particles larger than 100 nm are not similar, suggesting that the particle density at that diameter influenced filtration and deposition in the respiratory system of both rats and humans (Ji and Yu 2012).

This study characterised particles generated during the synthesis of AuNPs and AgNPs in a research laboratory in South Africa. The AuNPs and AgNPs released during the synthesis process were characterised based on particle size and number concentration. The number concentration was then used to derive mass concentration, which was used for exposure assessment and particle lung deposition, clearance and retention using the MPPD model. This study provides information on the mechanism for assessing external and internal exposure in a small-scale synthesis laboratory and the determination of the exceedance on the assigned or proposed OELs for AuNPs and AgNPs, respectively.

## 2 Materials and Methods

In this section, a detailed methodology used during the monitoring, characterisation, and exposure assessment is outlined. This was an experimental study where various variables such as temperature and humidity were controlled. During monitoring, the temperature ranged from 21 to 25°C while the humidity was between 40 and 55%.

### 2.1 Study Area

The study was conducted in a research laboratory where several syntheses are performed. The laboratory is located in Randburg, north of Johannesburg (26.1438° S, 27.9952° E).

### 2.2 Description of the Material Synthesis Process

The laboratory synthesises several research materials. The materials are synthesised in a chemical reactor chamber fitted with an extraction hood. The hood is also fitted with localised exhaust ventilation, and the front of the hood is fitted with a manually operated glass sash.

#### 2.2.1 Synthesis of Citrate Capped Gold Nanoparticles

Gold nanoparticles (AuNPs) are synthesised using Turkevish method. In a conical flask, 2 ml of HAuCl_4_.3H2O is added to ∼200 ml of de-ionised water. The solution is then brought to boiling at 100°C, while stirring at 800 rpms. Then after, 2 ml of 3.3% (m/v) citrate is added, while stirring. The solution is then left to stir at 800 rpm at the same temperature, until an appearance of a wine-red colour. The change of colour of the solution from colourless to wine-red indicates the formation of gold nanoparticles capped with citrate molecules. The solution is then cooled to room temperature at ∼ 25 C.

#### 2.2.2 Synthesis of Citrate Capped Silver Nanoparticles

The same AuNP synthesis procedure is repeated for the synthesis of citrate capped silver nanoparticles. However, the HAuCl_4_ is substituted with Ag(I)Cl. A change of colour from clear to bright-yellow indicates the formation of citrate capped silver nanoparticles.

**FIGURE 1:**
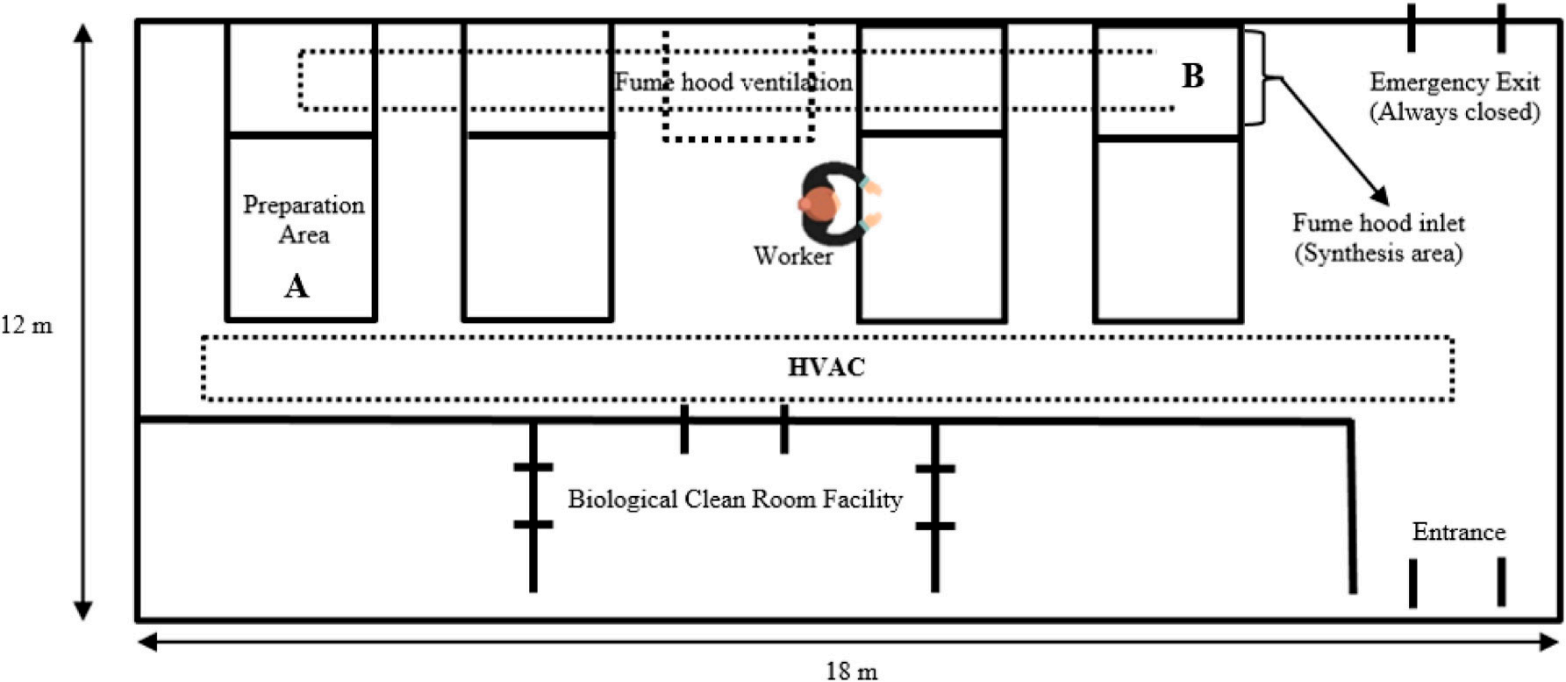
Schematic layout of the synthesis laboratory fitted with a heating, ventilation and air conditioning system (HVAC). C. Description of the ventilation systems.

A diagram of the laboratory layout is shown in Figure 1. The synthesis process is conducted in a glass vessel placed in a fume hood and is based on a process that includes heat, chemical reaction, and cooling. The raw unprocessed AuNPs and AgNPs are prepared on a preparation desk (A), on the side of the extraction hood. Once the materials are prepared, the synthesis is conducted in an enclosed hood (B), fitted with an extraction fan.

The laboratory is fitted with two mechanical ventilation systems i.e. HVAC system which supplies cold or warm air in the room and localised exhaust ventilation (LEV) fitted to the hood, aiding in extracting emissions arising from the synthesis process to the outside. In addition, to the mechanical ventilation system, the laboratory is fitted with two openable windows (800 mm-length x 110 mm-width). However, during synthesis the natural ventilation via opening windows is not used, but only HVAC and LEV systems are switched on. Despite the HVAC system being fitted with filters, there is a possibility of nanoparticles resulting from ambient air infiltrating into the indoor micro-environment. However, this study did not interrogate the contribution of ambient air nanoparticles into the synthesis laboratory. Therefore, there is a great need to investigate this contribution in future studies.

### 2.3 Data Collection Criterion

**FIGURE 2:**
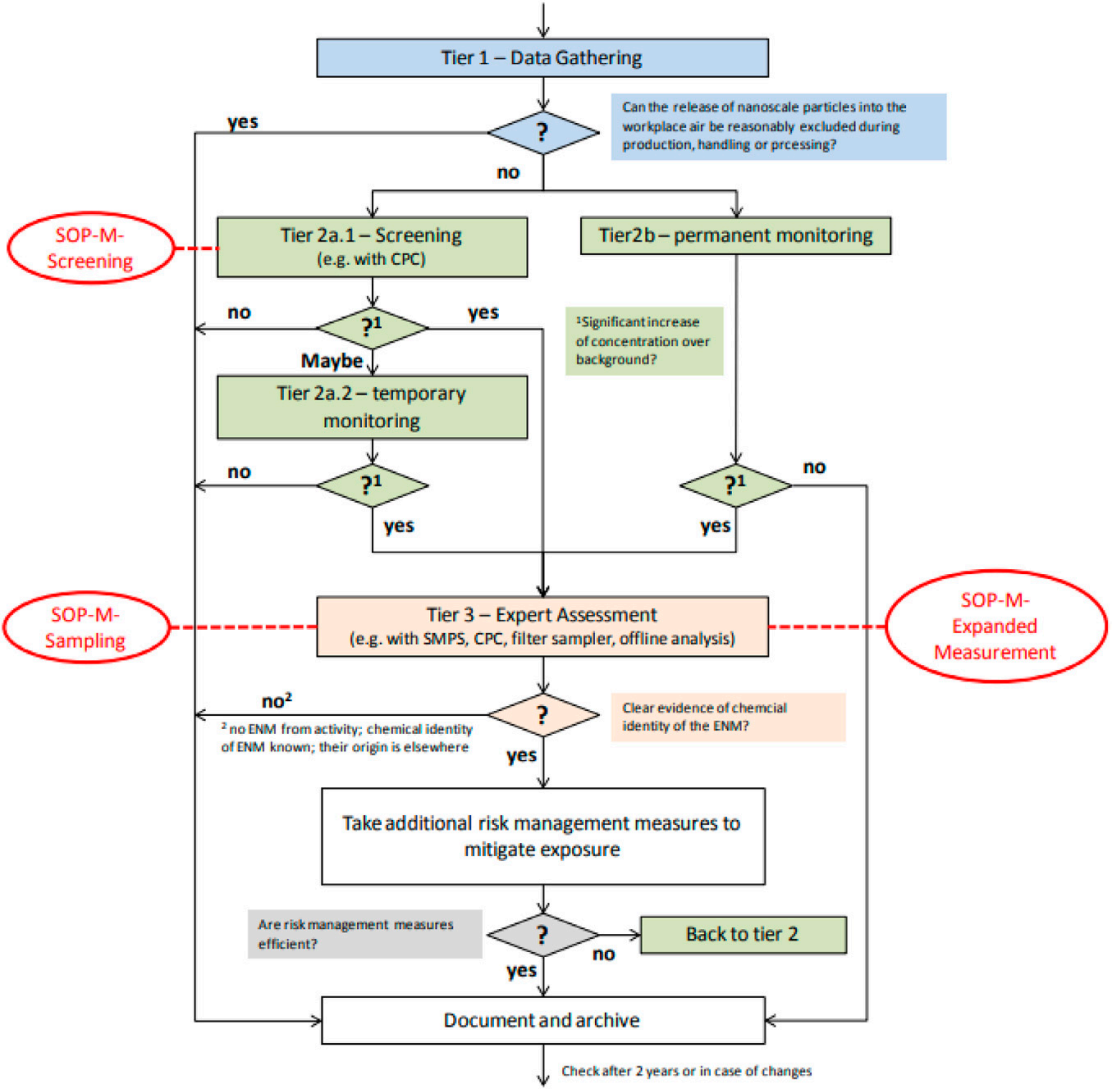
Tiered approach process flow (Kuhlbusch et al. (2012)).

This study used a 3-tiered stage measurement as described in details by Kuhlbusch et al. (2012) to correctly collect and characterise nanoparticles emitted during the synthesis process of AuNPs and AgNPs. A tiered approach is widely used in the literature due to the complexity of exposure assessment needs and is also known to be cost-effective. A simple demonstration of processes followed during the tiered approach is shown in Figure 2.

In tier one, an on-site inspection of the workplace was conducted to determine the potential release of ENPs during the synthesis processes. During the walkthrough survey observations, synthesis methodology and layout plans were scrutinised to understand the exposure pathway. Tier two followed the walkthrough survey, where detailed information was obtained on the sequence and location of each step during the process and also to determine time-activity patterns and understand particle release mechanisms. Then, in tier three, a series of monitoring and sampling was conducted to obtain information on particle number concentration.

### 2.4 Description of the Exposure Scenario

During a walk-through survey, it was discovered that the laboratory synthesises either Au or Ag material once a day. The synthesis process takes approximately 60 min from start to finish. Possible exposure may occur due to leakages from the reaction chamber, during stirring, and when removing a final product from the hood. The exposure duration was therefore determined to be 60 min, translating to the total time it takes to complete the synthesis process. However, the synthesis frequencies differ based on the amount of synthesis required for each day. Three exposure scenarios were therefore anticipated during the exposure concentration calculation for OEL comparison. Scenarios 1 (minimum exposure), 2 (moderate exposure) and 3 (worst-case) relate to one, three and five syntheses conducted on a given day, respectively, which translates to exposure durations of 60 min (Scenario 1), 180 min (Scenario 2) and 300 min (Scenario 3).

### 2.5 Gold and Silver Nanoparticles Monitoring

Similar to Brenner, (2016), we used a real-time aerosol instrument to monitor the ENPs during the synthesis processes. The ENPs were monitored continuously using a NanoScan SMPS Model 3910 (TSI Inc., Shoreview, MN, United States). A SMPS is a real-time monitor that logs data points continuously at a time interval of 60 s. A SMPS consists of built-in software; an electrostatic particle classifier, hardware system control and a condensation particle counter that performs analyses.

A particulate matter_1_ (PM_2.5_) cyclone with a 50% PM_1_ cut-off point was used to prevent larger particles from entering the SMPS. The instrument is capable of detecting particles ranging from 10–420 nm. Moreover, the instrument can measure concentrations as low as 100 particles/cm^3^ to a maximum of 10^6^ particles/cm^3^. Particles were classified into thirteen different size bins ranging from 11.5 to 365 nm. All measurements were done using a sheath flow rate of l L/min and a scan time of 60-s intervals. The instrument response time is 3 seconds but averages size distribution over 60-s intervals (45 s upscan and 15 s downscan).

### 2.6 Data Analysis

Data obtained from the NanoScan SMPS was exported to Microsoft Excel (Redmond, Washington, United States) for further analysis. The background concentration was calculated using Eq. 1 (Kuhlbusch et al. (2012)).

$$C_{BI}=\frac{1}{n}\sum_{k=1}^{n}C_{BkI}$$ (1)

Where C_BI_ is the average background concentration, *n* is the total number of measurements, and C_BKI_ is the background determination measurements.

The total particle concentration from the synthesis processes was calculated using Eq. 2.

$$C_{EI}=\frac{1}{n}\sum_{k=1}^{n}C_{EkI}$$ (2)

Where C_EI_ is the average emission concentration, *n* is the total number of measurements, and C_EKI_ is the emission determination concentration.

Since there will always be the presence of background concentration, Eq. 3 was used to obtain the net emission concentration (C_NET_).

$$C_{NET}=C_{BI}-C_{EI}$$ (3)

Where C_NET_ is the final corrected concentration resulting from a synthesis process, C_BI_ is the average background concentration, and C_EI_ is the average concentration from a synthesis process plus the background concentration.

### 2.7 Conversion of Particle Number Concentration to Mass

Since data obtained from the SMPS is expressed in number concentration, we converted number concentration to mass concentration to compare with the OEL for AgNPs (0.19 μg/m^3^), which is expressed in mass concentration (Hinds et al., 2001). In deriving the final concentrations, the background concentration was deducted from the post concentration. The post concentration was the concentration measured during the synthesis process, assuming that the background concentration is simultaneously measured. The conversion of particle number to mass concentration was only done using the total count concentration after the background total count number concentration was deducted as in Eq. 4. Since, the data was acquired using a SMPS Eq. 4 was used to convert number concentration to mass concentration, because it is assumed that the SMPS bin diameter corresponds to the geometric diameter (Fissan et al., 2014). For the background concentration the particle density was assumed to be 1.2 g/cm^3^ (Cross et al., 2007).

$$C_{m}=\sum F_{C}\rho pC_{Ni}\frac{\rho \pi }{6}di^{3}$$ (4)

Where Cm is the calculated mass concentration, Fc is the particle unit conversion factor (10^−15^), *ρp* is the particle density (g/cm^3^), C_Ni_ is the derived SMPS number concentration (#/cm^3^), ρπ is the ratio of the circumference of any circle to the diameter of that circle (3.142) and di is the averaged midpoint of the size bin. In calculating the final mass concentration, data were extracted from the SMPS and transferred to an excel sheet where particle number concentration, particle density (1.2 g/cm^3^), and median diameter were retrieved. We further calculated an 8-h time-weighted exposure concentration for AuNPs and compared it with the proposed OEL for AuNPs (20,000 particles/cm^3^).

### 2.8 Particle Lung Deposition Estimation

In this study, the MPPD model (version 3.04, Chemical Industry Institute of Toxicology, Research Triangle Park, NC; https://www.ara.com/mppd/) was used to estimate the particle fate in the human lung respiratory system. The MPPD model calculates the deposition of ENPs between 1 nm and 100 µm and the retention of particles in the respiratory tract. Although most of the parameters used in the MPPD model are from rats, a systematic extrapolation of rat to human data is deemed a more realistic prediction mechanism (Ginsberg et al., 2008; Asgharian 2018). Despite differences in the lung geometry of the rat and human lung, the MPPD model is utilised in various settings to communicate the risk of exposure to several compounds (Ji and Yu 2012; Buckley et al., 2016; Kim et al., 2020). Table 1 shows the parameters used in the MPPD model.

**TABLE 1:** Assigned values used in the MPPD modelling.

Model Parameter	Human (Low Exertion)
a) Lung morphology	
⁃ Model	Yeh/Schum 5 lobe, Ji and Yu (2012)
⁃ FRC (functional residual capacity)	3300 ml (default)
⁃ URT (upper respiratory tract)	50 ml (default)
⁃ Surface area of the alveolar region	62.7 m^2^, Oller and Oberdörster (2010)
b) Particle properties (silver and gold)	
⁃ Particle density	10.49 g cm^−3^ (silver); 19.3 g/cm^3^ (gold)
⁃ GM	52.6 nm (silver); 26.1 nm (gold)
⁃ GSD	2.0 (silver); 2.2 (gold)
c) Exposure conditions	
⁃ Concentration	0.047 μg/m^3^ (gold); 0.077 μg/m^3^ (silver)
⁃ Breathing frequency	1024, Ji and Yu (2012)
⁃ Tidal volume	20 per minute
⁃ Exposure hours/day	1 h/day
⁃ Exposure days/week	5 days/week
⁃ Exposure weeks	48 weeks (4 weeks leave)
d) Clearance settings	
⁃ Fast human clearance	0.02 per day (default value)
⁃ Medium clearance	0.001 per day (default value)
⁃ Slow clearance rate	0.0001 per day (default value)

### 2.9 Quality Control

Before sampling, the benches and fume hood were cleaned using 70% ethanol to remove any particles and other residual materials. Throughout the monitoring campaign, doors and windows leading to the outside were all closed to prevent the intake of outdoor incidental particles. Thereafter, access was not allowed to the laboratory to avoid the exchange of ambient air carrying incidental materials into the laboratory. An annual instrument calibration as per the manufacturer’s instruction was done by a South African National Accreditation System (SANAS) accredited laboratory. A span calibration followed by a zero calibration was performed before each monitoring campaign. Since submicron particles are always present in the laboratory, the background concentration was monitored to account for non-process-based particles during the evaluation. The SMPS was allowed to run for a 45-min interval to collect background particle number concentration before the synthesis process could begin.

## 3 Results and Discussion

### 3.1 Gold Nanoparticles

Table 2 presents the background concentration and actual synthesis-based particle number concentration results. For the background, particles in the Accumulation and Aitken mode were greater than the actual synthesis process. The measured background particles might be due to resuspension caused by an increase in indoor air velocity and turbulence created by human movement in the laboratory. Since all doors and windows were closed, the settled particles may be from leakages from the hood and others through infiltration from the outdoor. Notably, the measured particle modes were mostly dominant in the Nuclei mode during the background and synthesis, while the Accumulation mode recorded the lowest percentage contribution during the Au synthesis process.

**TABLE 2:** Particle mode percentage contribution accounting for both background and gold synthesis process.

Background Concentration		
Particle Mode	#/Concentration	%Contribution
Nuclei (<50 nm)	8266.1	68.1
Aitken (50–100 nm)	2,310.7	19.0
Accumulation (>100 nm)	1567.4	12.9
Synthesis (Gold)		
Nuclei	10,882.4	79.1
Aitken	1612.1	11.7
Accumulation	1263.9	9.2

From Table 2, it can be observed that most nanoparticles generated during the synthesis of Au are in the Nuclei mode, which accounted for 79.1%. This finding suggests that the particles were emitted as single particles and did not undergo a physical transformation. Although there is a slight increase in the ambient measured concentration of nanoparticles, there is little difference in terms of the number concentration compared to the background. This finding was expected since the synthesis process was performed inside an enclosed hood fitted with a localised exhaust ventilation system.

**FIGURE 3:**
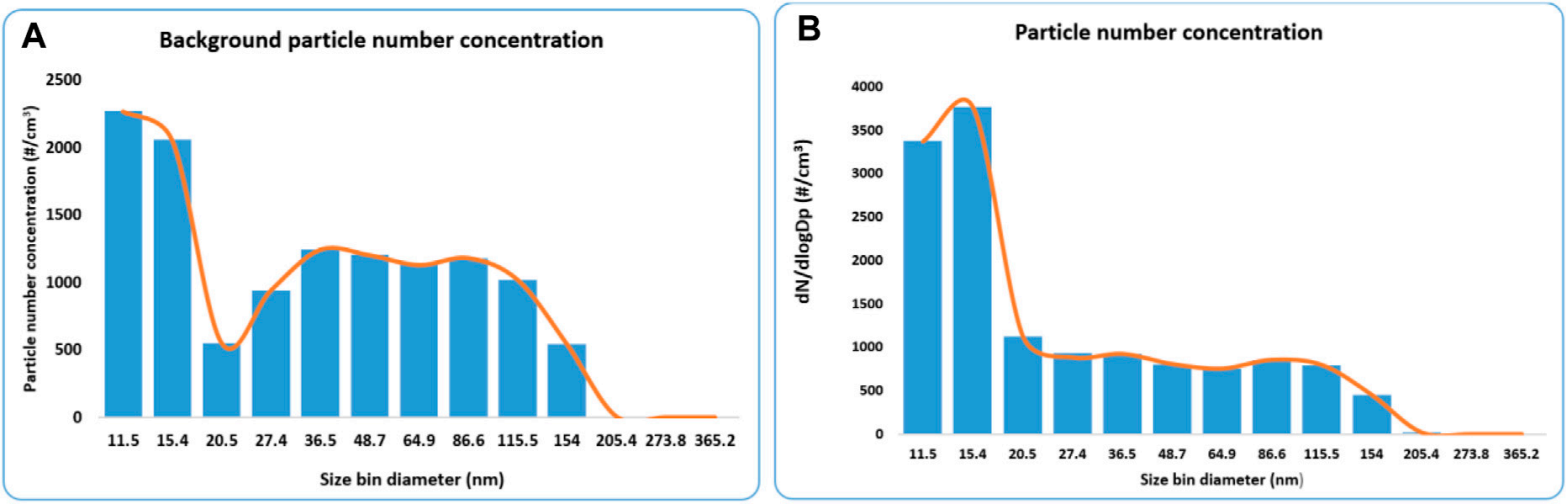
**(A)** Particle number concentration for the background in the gold synthesis laboratory and **(B)** Particle number concentration for the gold synthesis process.

Figures 3A,B shows the particle number concentration according to the size bin for both the background and synthesis process of AuNPs. The two size bins (11.5 and 15.4) recorded the highest number of particles, suggesting that most particles are below 20 nm. However, for the background concentration, the particle mode was approximately 50 nm, while for the synthesis process, the mode was around 13.8 nm. This finding is concerning since it has been established that the toxicity of ENPs increases with a decrease in size (Boyoglu et al., 2013; Smith et al., 2018; Ul-Hamid 2018).

The measured particle number concentration was averaged over 1-h corresponding to the synthesis process duration for the triplicated monitoring campaigns. The derived concentrations were averaged and presented using two exposure assessment metrics (number concentration and mass concentration), as shown in Table 3. A particle mode of 13.8 nm was found during the AuNPs synthesis process. The particle mode or diameter is important in future toxicity assessments either during *in vitro* or *in vivo* assays (Anwar and Halim 2012; Vetten et al., 2013).

**TABLE 3:** Gold nanoparticles emission data averaged over an hour (*n* = 3).

Sample Item	Concentration ±SD	Concentration	Median	Mean	GM	Mode	GSD
	#/cm^3^	µg/m^3^	nm	nm	nm	nm	
Background	12,141.2 ± 125	0.348	30.6	44.1	31.0	50.8	2.1
Gold synthesis	13,758.4 ± 102	0.395	19.7	37.7	26.1	13.8	2.2
Final concentration	1617.3 ± 109 (11.8%)	0.047	—	—	—	—	—

Table 4 presents results for the three exposure scenarios used to derive an 8-h equivalent exposure concentration during the synthesis of AuNPs. For the worst-case scenario, the exposure to AuNPs was 19.6-fold lower than the NRV for AuNPs. In the moderate and minimum exposure scenarios, the derived 8-h equivalent concentration was 33 and 99 folds lower than the NRV for AuNPs, respectively.

**TABLE 4:** An 8-h equivalent exposure concentration compared against proposed provisional nano reference value for gold.

Exposure Scenario	Exposure Duration (Minutes)	TWA_8equivalent_ Concentration (#/cm^3^)	Proposed Provisional Reference (20,000 #/cm^3^)
Scenario 1 (minimum)	60	202.2	< proposed OEL
Scenario 2 (moderate)	180	614.6	< proposed OEL
Scenario 3 (worst-case)	300	1018.9	< proposed OEL

### 3.2 Silver Nanoparticles

Table 5 presents the AgNPs results together with the background concentrations. Three different particle modes were found, however, there were fewer particles in the Accumulation mode relative to the Nuclei and Aitken mode. From Table 5, it can be further observed that particles in the Nuclei mode were dominant, accounting for over 50% of the total number concentration. In the Accumulation mode, the % contribution of the particles for the background concentration was higher than the synthesis process. This finding suggests that larger particles monitored during the synthesis process were mainly from background suspensions in the synthesis laboratory. The variability in the background concentrations, which were at times being higher than the synthesis emitted particles were also reported elsewhere (Methner et al., 2010).

**TABLE 5:** Particle number concentration and particle mode for the background and silver synthesis process.

Background Concentration		
Particle Mode	#/Concentration	%Contribution
Nuclei (<50 nm)	3789.5	51.6
Aitken (50–100 nm)	2,109.9	28.7
Accumulation (>100 nm)	1439.6	19.6
Synthesis (Silver)		
Nuclei	5364.7	53.5
Aitken	2,916.0	29.1
Accumulation	1746.0	17.4

**FIGURE 4:**
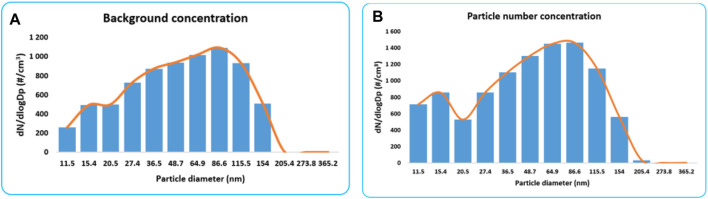
**(A)** Particle number concentration for the background and **(B)** particle number concentration for the silver synthesis process.

Figure 4A,B shows the results of the background concentration as well as the silver synthesis. The particle number concentration for the background is higher for the Aitken mode compared to accumulation mode. Particle size bins between 36.5 and 115.5 recorded the highest number of particles, representing a different mode compared to the gold synthesis process (Figure 3B).

AgNPs were averaged over 1-h, where particle mode and geometric mean and standard deviations are presented in Table 6. The average concentration derived using triplicate measurements showed a 1-h concentration of 0.077 μg/m^3^, with a particle mode of 80.8 nm. This finding introduces additional important information which is likely to be used in laboratory testing where toxicity assays will be conducted. Several *in vitro* studies have assessed the toxicity of AgNPs using diameters below 20 nm (Stensberg et al., 2011; Riaz Ahmed et al., 2017). Therefore, it may be of interest to consider the actual diameter of emitted particles in toxicity assessment when trying to replicate field-based practices.

**TABLE 6:** Silver nanoparticles emission data averaged over an hour (n = 3).

Sample Item	Concentration ±SD	Concentration	Median	Mean	GM	Mode	GS
	#/cm^3^	µg/m^3^	nm (#/cm^3^)	nm (#/cm^3^)	nm (cm^3^)	nm (cm^3)^	nm
Background	7339.1 ± 403	0.211	48.9	58.9	44.5	78.4	2.2
Silver nanoparticles synthesis	10,026.7 ± 590	0.288	57.5	64.3	52.6	80.8	2.0
Final concentration	2,687.6 ± 620	0.077	—	—	—	—	—

In Table 7, the measured airborne concentration is compared with the occupational exposure limit for AgNPs for the three possible exposure scenarios. For the possible exposure scenarios, the concentrations to which employees are exposed are below the OEL. The worst-case scenario was four-folds below the OEL, while the moderate and minimum exposure scenarios were seven and 21 folds lower than the OEL, respectively.

**TABLE 7:** An 8-h equivalent exposure concentration compared to the OEL for silver nanoparticles.

Exposure Scenario	Exposure Duration (Minutes)	TWA_8equivalent_ Concentration (µg/m^3^)	OEL for Silver (0.19 μg/m^3^)
Scenario 1 (minimum)	60	0.009	< OEL
Scenario 2 (moderate)	180	0.029	< OEL
Scenario 3 (worst case)	300	0.048	< OEL

The background concentrations in the two laboratories are highly variable, with gold laboratory background concentration being characterised with higher particle concentrations especial in the two lowest size bins. The issue of background concentration in several laboratory and field studies has been flagged out in previous review publication (Kuhlbusch et al., 2011). Should our suspicion of the two size bin concentration be correct suggesting that an increase in number concentration be as a result of the previous gold synthesis process, this may imply that the exposure can be 15 times folds higher that what is currently reported. Therefore, a chemical analysis data can aid in clarifying this uncertainty, though it is not an easy task to perform given a lower particle filter loading for this type of synthesis often leading to failure to be detected by a laboratory analytical method.

In summary despite the exposure risk being estimated to be low, it must be noted that the risk of exposure for this study was only based on emissions during the synthesis process without emissions from material preparations and post synthesis which takes place outside the hood. Published literature suggests that elevated exposures may be expected during material preparation and post treatment (Lo et al., 2011). However; the type of materials synthesised (Nano powders) and post treatment activities such as opening the reactor, removal of the insulator, filtration and material cooling could lead to elevated background levels (Kuhlbusch et al., 2011; Shaughnessy 2013). Therefore, it remains important for future prospects exposure assessments studies to assess the contribution of material preparation and post treatment emissions to the exposure risk quantification process.

### 3.3 Multiple-Path Particle Dosimetry Model Data for Silver and Gold Nanoparticles

**FIGURE 5:**
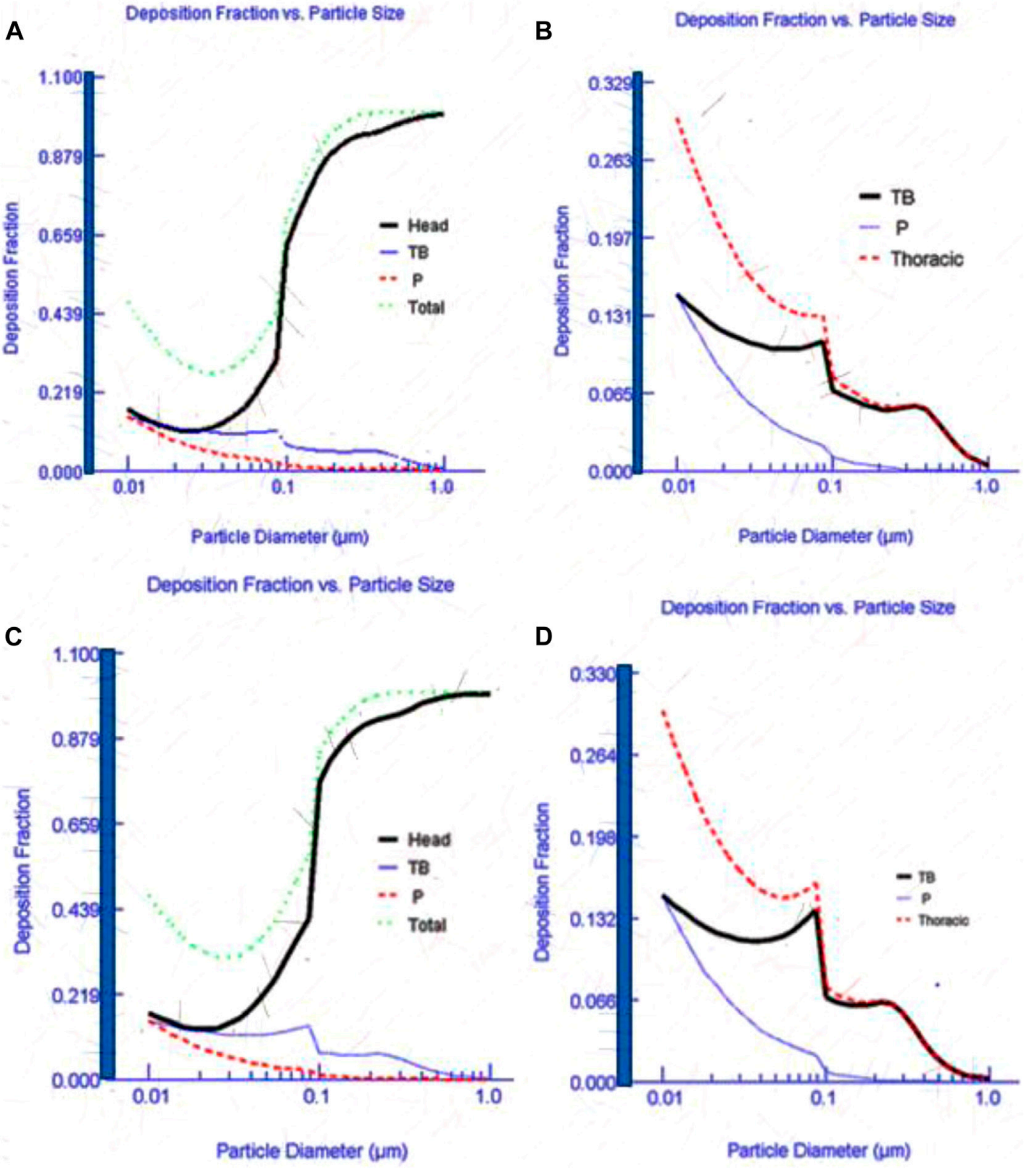
Deposition fraction of Au **(A–B)** and Ag **(C–D)** nanoparticles in a simulated human respiratory system.

MPPD model deposition efficiencies using input parameters shown in Table 1 are illustrated in Figure 5. Figure 5A,B (AuNPs) and c-d (AgNPs) shows the fraction of emissions produced during the synthesis process and deposited into a simulated human respiratory system. Most of the particles below 100 nm were deposited in the pulmonary region followed by the tracheobronchial and head region. A small fraction of particles deposited in the head region. It has been established that ENPs tend to deposit deeper into the lungs while submicron particles deposit in the bronchial region. Therefore, this finding was expected since most of the synthesis-based particles were dominated by particle mode of around 80 nm. A similar deposition was reported in other studies (Oller and Oberdörster 2010; Ji and Yu 2012; Buckley et al., 2016). Particle deposition in the head region increases as the particle diameter increases. Therefore, the current practice of assessing occupational exposure by monitoring respirable particles may be irrelevant in synthesis laboratories since most of the particles are in the nano-range.

**FIGURE 6:**
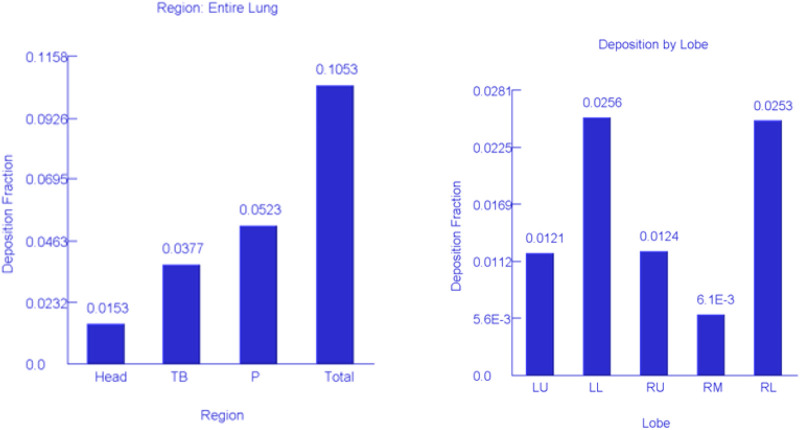
Simulation of lung deposition for the entire lung and lobes. Head, head region; TB, tracheobronchial region; P, pulmonary region; LU, left upper lobe; LL, left lower lobe; RU, right upper lobe; RM, right middle lobe; RL, right lower lobe.

Using the MPPD model, three different particle deposition mechanisms (sedimentation, diffusion, and interception) were incorporated into the modelling process (Figure 6). Figure 6 indicates that over 50% of the particles were deposited in the pulmonary region of the lung while 38 and 15% were deposited in the tracheobronchial and head region, respectively. The deposition of particles in the head region might likely be a contribution of the larger particles >100 nm.

### 3.4 Multiple-Path Particle Dosimetry Model Data for Gold and Silver Nanoparticles Clearance and Retention

**FIGURE 7:**
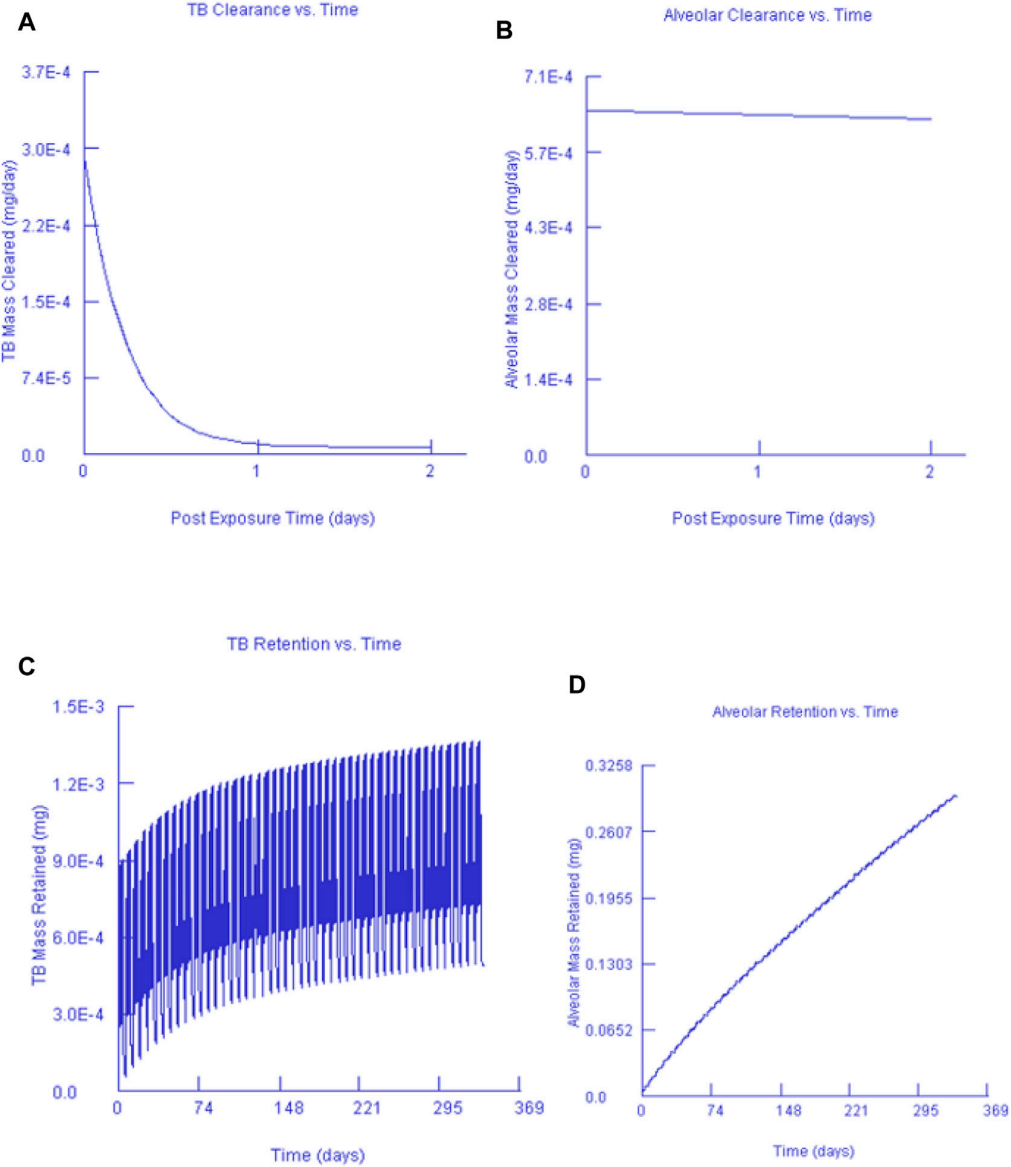
Gold nanoparticles and their corresponding TB clearance **(A)** and alveolar clearance **(B)** for simulated post-exposure as well as their TB retention **(C)** and alveolar retention **(D)** for simulated continuous exposure.

**FIGURE 8:**
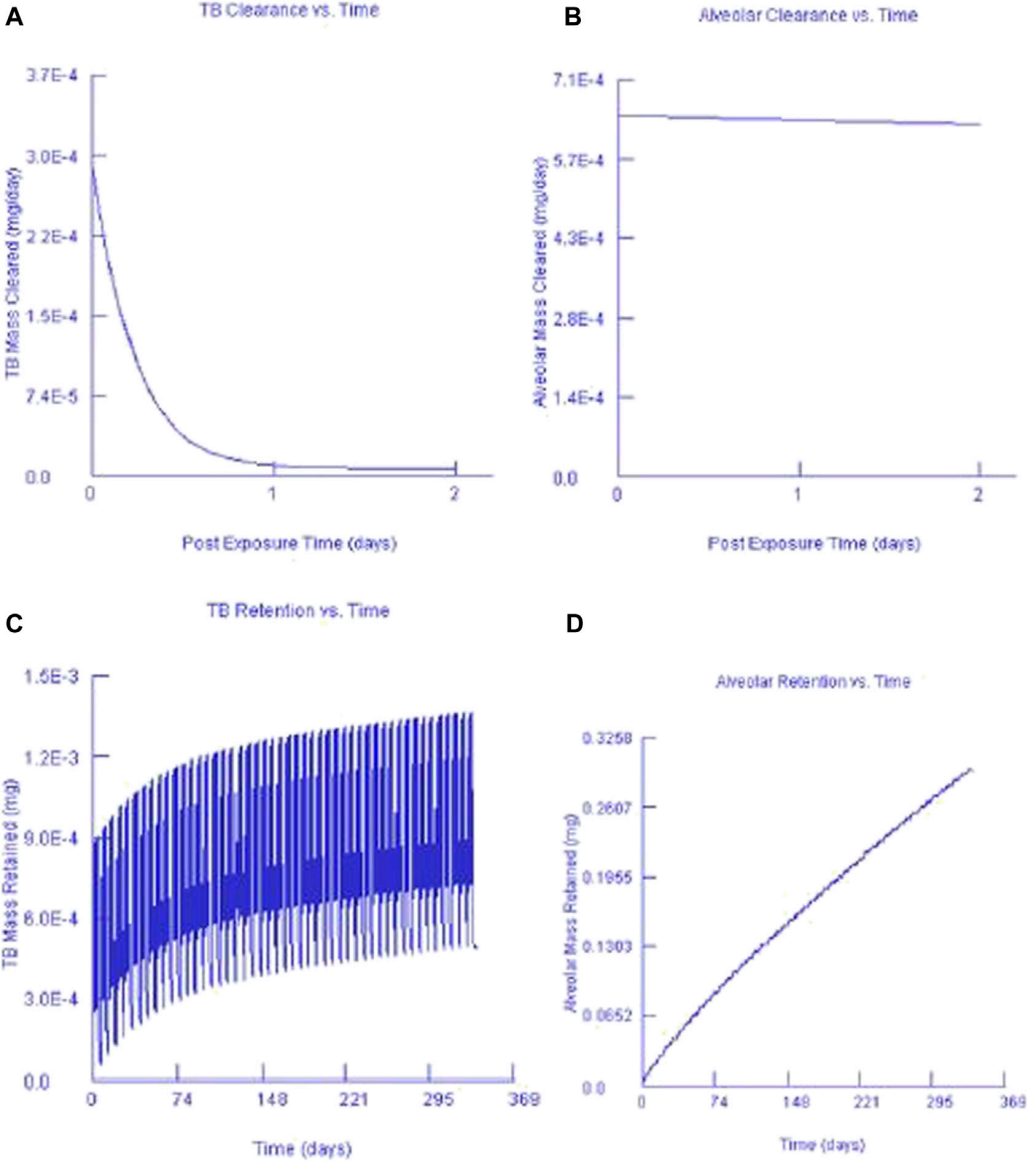
Silver nanoparticles and their corresponding TB clearance **(A)** and alveolar clearance **(B)** for simulated post-exposure as well as their TB retention **(C)** and alveolar retention **(D)** for simulated continuous exposure.

Figures 7, 8 show the clearance and retention for AuNPs and AgNPs simulated using the MPPD model. Both Au and Ag model output shows a similar clearance and retention mechanism. The tracheobronchial (TB) clearance in both Au and Ag indicated a fast removal mechanism lasting for about 2 days after exposure, while a longer clearance period was observed for the alveolar region. Similar observations were recently reported by Jo et al. (2020) and Kim et al. (2021) who found that AgNPs clearance occurs in fast mode. Jo et al. (2020) concluded that the fast clearance may be due to the dissolution of Ag from the AgNPs while the slow clearance is due to the poorly soluble AgNPs secondary particles from the Ag ions reacting with biogenic anions.

Our findings are in agreement with Anderson et al. (2015), that particle size affects macrophage clearance, however, it has an insignificant effect on the long-term retention of AgNPs in the lungs. In both instances, it can also be seen that only a small fraction of the deposited concentration is cleared while most of the deposited fraction is retained. This finding was expected since AuNPs and AgNPs do not metabolise once they are deposited. Our findings are in agreement with Anderson et al. (2015) who found that AgNPs can be retained in the terminal TB and alveolar region duct irrespective of the particle size.

## 4 Conclusion

This study has characterised emissions from the gold and silver synthesis process in a research laboratory. A methodology for converting particle number concentration to mass concentration was presented for the first time in the South African context. The particles emitted from both Au and Ag synthesis indicated a particle mode of around 13.8 and 80.8 nm, respectively. The 8-h equivalent exposure concentration was below the proposed provisional reference value for AuNPs. Also, exposure to AgNPs was below the occupational exposure limit. This finding suggests that workers in the research laboratories may not be at risk of elevated exposures during the synthesis process. Therefore, it can be recommended that workers in this setting can continue working without any personal protective clothing where inhalation is determined to be the only route of entry. This finding was expected since the literature suggested that the use of localised exhaust ventilation significantly reduces the exposure intensity of ENPs (Methner et al., 2010). Furthermore, the synthesis process was conducted in an enclosed hood, therefore the emission was anticipated to be from leakages on the hood or escaping during the opening of the hood door. For human respiratory model output, we have found that over 80% of the deposited fraction was in the TB and alveolar region. In addition, there was a poor particle clearance at the alveolar region compared to the TB region. Higher particle retention was also found at the TB and alveolar region. This finding is concerning, given that once ENPs are deposited in the lower respiratory tract they do not metabolise, which may lead to severe chronic health consequences. Although, an 8-h equivalent OEL was not exceeded, workers may still be at risk of associated effects due to particle accumulation as a result of a higher particle retention and a lower particle clearance in the lower respiratory tract. Our finding are similar to the study published focusing on particle deposition, retention and clearance of inhaled ENPs (Lee et al., 2012).

## Data Availability

The raw data supporting the conclusions of this article will be made available by the authors, without undue reservation.
